# Sticky Bacteria: Understanding the Behavior of a D-Galactose Adapted Consortium of Acidophilic Chemolithotroph Bacteria and Their Attachment on a Concentrate of Polymetallic Mineral

**DOI:** 10.3389/fmicb.2021.767639

**Published:** 2021-10-21

**Authors:** Paulina Aguirre, Albert Saavedra, Eduardo Moncayo, Sabrina Hedrich, Karlo Guerrero, Juan Carlos Gentina

**Affiliations:** ^1^Departamento de Química, Universidad Técnica Particular de Loja (UTPL), Loja, Ecuador; ^2^Escuela de Ingeniería Bioquímica, Pontificia Universidad Católica de Valparaíso, Valparaíso, Chile; ^3^Institute of Biosciences, TU Bergakademie Freiberg, Freiberg, Germany

**Keywords:** bacterial attachment, D-galactose, *At. thiooxidans*, *L. ferrooxidans*, biooxidation

## Abstract

Various strategies to accelerate the formation of biofilms on minerals have been studied, and one of them is the use of D-galactose as an inducer of EPS production in planktonic cells of biooxidant bacteria. With the aim to evaluate the influence on the attachment and the effect over the solubilization of a polymetallic mineral concentrate, the behavior of a microbial consortium formed by *Acidithiobacillus thiooxidans* DSM 14887^T^ and *Leptospirillum ferrooxidans* DSM 2705^T^ previously induced with D-galactose for the early formation of EPS was studied. These microorganisms were previously adapted to 0.15 and 0.25% of D-galactose, respectively; afterward, different proportions of both strains were put in contact with the particle surface of a concentrate of polymetallic mineral. Also, to evaluate the affinity of each bacterium to the mineral, attachment tests were carried out with one of these species acting as a pre-colonizer. The same consortia were used to evaluate the solubilization of the polymetallic mineral. The results obtained show that the induction by D-galactose increases the microbial attachment percentage to the mineral by at least 10% with respect to the control of non-adapted consortia. On the other hand, the tests carried out with pre-colonization show that the order of inoculation also affects the microbial attachment percentage. From the different proportions tested, it was determined that the use of a consortium with a proportion of 50% of each species previously adapted to D-galactose and inoculated simultaneously, present a microbial attachment percentage to the mineral greater than 95% and better solubilization of a polymetallic mineral, reaching values of 9.7 and 11.7mgL^−1^ h^−1^ of Fe^3+^ and SO_4_^2−^, respectively. Therefore, the use of D-galactose in small concentrations as inducer of EPS in acidophilic cells and the selection of an adequate strategy of inoculation can be beneficial to improve biooxidation since it would allow this process to develop in a shorter time by achieving a greater number of attached cells in a shorter time accelerating the solubilization of a sulfide mineral.

**Graphical Abstract:**
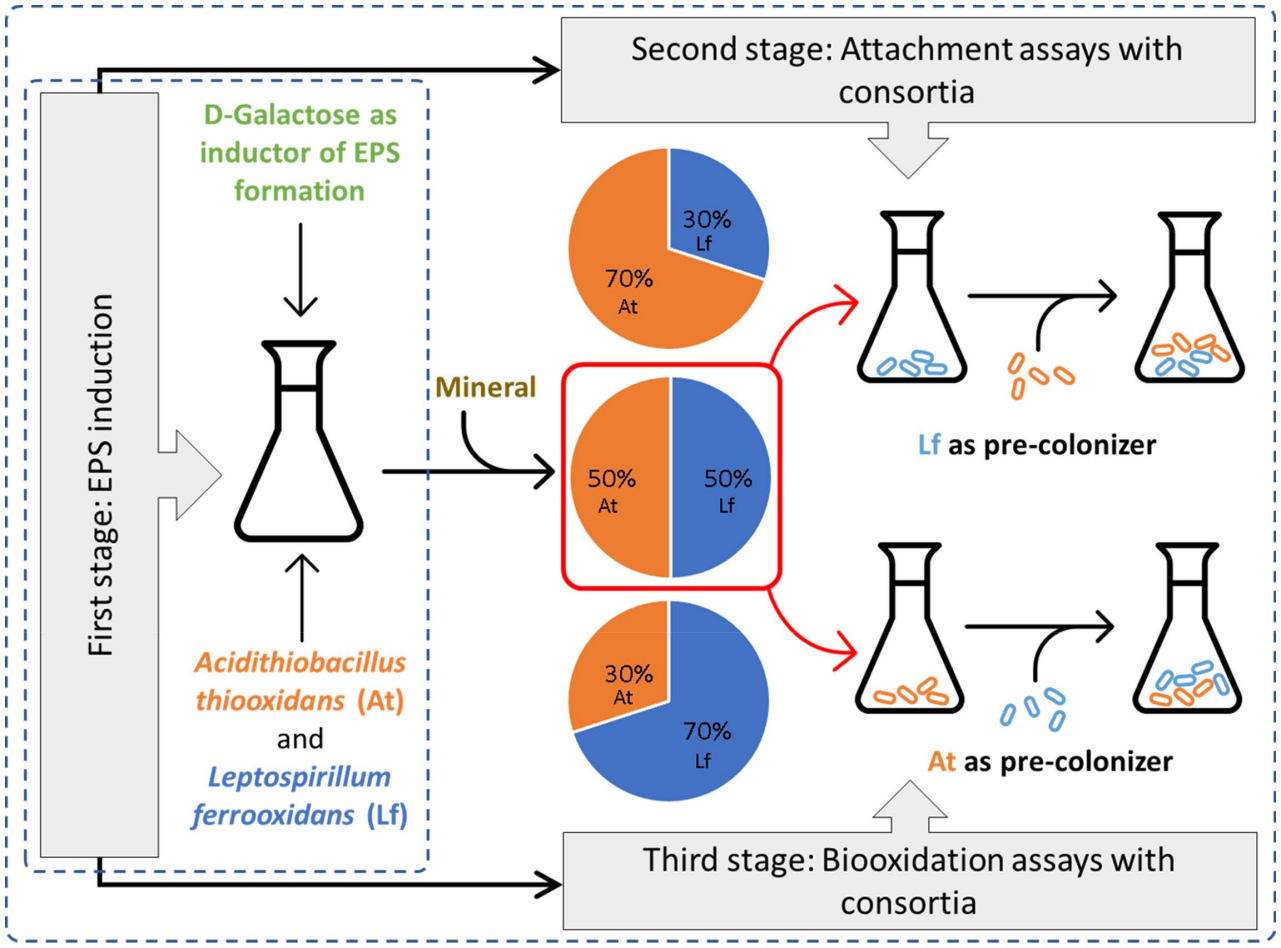
EPS production using D-galactose as inducer and its influence in the attachment of consortia formed by differents proportions of *A. thiooxidans* and *L. ferrooxidans* inoculated at the same time and when one of them acting as a pre-colonizer.

EPS production using D-galactose as inducer and its influence in the attachment of consortia formed by differents proportions of *A. thiooxidans* and *L. ferrooxidans* inoculated at the same time and when one of them acting as a pre-colonizer.

## Introduction

Biooxidation processes have become increasingly important for the recovery of valuable metals from low-grade minerals, where conventional processing is not feasible (Gentina and Acevedo, 2016). Chemolithoautotrophic bacteria, such as *Leptospirillum (L.) ferrooxidans* and *Acidithiobacillus (At.) thiooxidans* (Nemati and Harrison, 2000), are used for this purpose since they are capable of oxidizing iron and sulfur compounds, respectively, to obtain energy. During this process, bacteria play an important role in biooxidation through the regeneration of ferric iron (Fe^3+^) and the production of sulfuric acid. When they attach to the mineral and oxidize the ferrous ion (Fe^2+^), they generate ferric iron (Fe^3+^) which solubilizes the sulfur minerals in an acidic environment where the metals of interest are occluded; this occurs at the interface of the extracellular polymeric substances (EPS) produced by microorganisms and the mineral. This has been used, as an example, to release gold from sulfide matrix and its subsequent leaching with cyanide (Vera et al., 2013; Saavedra et al., 2020).

The attachment of microorganisms to the surface of minerals is a key step for the biooxidation process to take place (Bellenberg et al., 2014a). During this stage, microorganisms are organized into complex structures called biofilms (Lara et al., 2010). In a biofilm, the microorganisms are embedded in EPS that constitute a biochemical reaction space for biooxidation, which occurs in the microenvironment generated by the biofilm and the Fe^3+^ ions that are located between the cell walls of the microorganisms and the surface of metal sulfides (Sand and Gehrke, 2006).

In the case of biooxidant microorganisms, biofilm formation allows them to bind to the mineral surface through electrostatic forces and hydrophobic interactions, thus facilitating the oxidizing action of Fe^3+^ on metal sulfide (Karatan and Watnick, 2009; Aguirre Morales, 2012). In turn, EPS play an important role in the initial stages of the mineral attachment process and biofilm formation (Gehrke et al., 1998; Sand et al., 2001). Subsequently, biooxidant microorganisms which do not form EPS need a longer time to attach to the mineral surface and consolidate the biofilm. In addition, attachment rates are lower for cells that previously formed EPS (Arredondo et al., 1994; Saavedra et al., 2013). Therefore, microbial attachment and subsequent biofilm formation on the mineral surface play a fundamental role in the biooxidation processes of minerals (Rodríguez et al., 2003). In this regard, attachment strategies based on the induction of EPS have been proposed as an adaptive response to substances that present toxicity such as D-galactose (Silver et al., 1967; Barreto et al., 2005).

On the other hand, it has been observed that there are changes in the composition of the microbial communities present in the biooxidation processes; however, the interactions among the different biooxidant species that coexist in the biofilms formed on sulfur minerals are generally unknown (Bellenberg et al., 2014a). It has been shown that biofilms from different ecological habitats suffer a succession of colonizers where the former produces EPS that can promote the attachment of other species (Elifantz et al., 2013).

According to Castro et al. (2016), the attachment of the cells and the subsequent formation of the biofilm depends on both, the substrate and the species involved in the process. These researchers, in their work with pre-colonization coupled with the inoculation of a second species to generate a consortium of *Acidianus* spp. and *Sulfolobus metallicus,* suggest that both species mutually influence each other with respect to the initial adherence speed. Similarly, Liu et al. (2016) showed the existence of interspecies interactions during the initial attachment of a model consortium formed by *Sulfobacillus thermosulfidooxidans* DSM 9293^T^ and *Acidianus* sp. DSM 29099. The work of Rawlings and Johnson (2007) has shown that mixed cultures are more efficient in the bioleaching process than pure cultures as they improve the robustness of bioleaching and resistance to disturbances. A set of relevant microbial populations could potentially reduce the operational cost in bioleaching and biooxidation processes, which would otherwise occur when unwanted microbial communities are present (Rawlings and Johnson, 2007). Thus, Falco et al. (2003) developed covellite bioleaching tests showing that mineral solubilization performed better with a microbial consortium including *At. thiooxidans* and *L. ferrooxidans*.

Given the fact that various authors have shown that for bioleaching bacteria the attachment speed depends on factors such as the substrate, the species involved, the production of EPS, among others (Harneit et al., 2006; Kock and Schippers, 2008; Yang et al., 2015), it is important to study the microbial behavior in terms of attachment percentage to the mineral and their performance when they are established as a consortium. By virtue of these antecedents, the objective of this work was to evaluate the behavior of a model microbial consortium containing the bacteria *At. thiooxidans* DSM 14887^T^ and *L. ferrooxidans* DSM 2705^T^ previously adapted to D-galactose for the early formation of EPS and their influence on the attachment percentage and subsequently their effect over the solubilization of iron and sulfur from a concentrate of polymetallic mineral.

## Methodology

### Microorganisms

The microorganisms used were *At. thiooxidans* DSM 14887^T^ and *L. ferrooxidans* DSM 2705^T^. Cultivation was carried out in 250-ml Erlenmeyer flasks containing 50ml of a culture medium with the following composition in g·L^−1^: KCl 0.004, MgSO_4_·7H_2_O 0.005, (NH_4_)_2_HPO_4_ 0.15, CaCl_2_ 0.0012 (Kim et al., 2002), supplemented with 44g·L^−1^ FeSO_4_·7H_2_O for *L. ferrooxidans* DSM 2705^T^ or 10g·L^−1^ S° for *At. thiooxidans* DSM 14887^T^. The culture conditions were 30°C and 180rpm, at pH 1.8 and 1.3 for *At. thiooxidans* DSM 14887^T^ and *L. ferrooxidans* 2705^T^, respectively.

### Mineral

A polymetallic mineral concentrate was used for the attachment tests. The mineral was obtained from Bella Rica, Azuay Province (Ecuador). X-ray diffraction (XRD) analysis of the concentrate revealed the following composition in %: 30.23 quartz, 34.26 pyrite, 0.41 chalcopyrite, 0.95 galena, 3.41 sphalerite, 15.19 orthoclase, 8.24 gypsum, 0.89 apatite-(Sr-OH), 2.41 pyrrhotite, 2.61 arsenopyrite, 0.92 cobalt pentlandite, and 0.46 magnetite. The mineral used was crushed to a particle size of 75μm verified by the D80 of the ASTM # 200 sieve.

The mineral was acidified to pH 1.8 with a solution of 10N H_2_SO_4_, dried, and exposed to a N_2_ atmosphere to avoid chemical oxidation. Finally, it was sterilized at 121°C for 24h with dry heat (Bellenberg et al., 2014b).

### Adaptation of Microorganisms to D-Galactose

*At. thiooxidans* DSM 14887^T^ and *L. ferrooxidans* DSM 2705^T^ were previously adapted to 0.15%, 0.25, and 0.35% of D-galactose as described in Aguirre et al. (2017 , 2018). The maximum specific growth rate (μ_max_) and the yield of biomass (Y_X/S_) were calculated for each adapted strain (see Supplementary Material). The amount of EPS produced by each species was extracted using Dowex cationic exchange resin (CER DOWEX Marathon C, SIGMA) according to Bellenberg et al. (2014b), and then, it was quantified by measuring of its main components: proteins and carbohydrates. Protein concentration was determined by the Bradford method (Bradford, 1976) and carbohydrates by the phenol/sulfuric acid method (Dubois et al., 1956).

### Evaluation of the Microbial Attachment to the Mineral Surface

To evaluate the microbial attachment to the mineral surface, different consortia were formed with *At. thiooxidans* DSM 14887^T^ and *L. ferrooxidans* DSM 2705^T^ adapted to D-galactose, while non-adapted cells were used to form consortia that acted as control. Mixtures of both strains in proportions of 30:70, 50:50, and 70:30 simulated the different microbial consortia. In all cases, a total of 1·10^8^ cells·ml^−1^ were initially inoculated.

Experiments were carried out in 250-ml flasks containing 50ml of Kim medium (without addition of D-galactose, iron, and elemental sulfur) supplemented with 2% w/v of a polymetallic mineral concentrate and inoculated with a total of 1·10^8^ cells·ml^−1^ in accordance with the consortia established before. For the evaluation of the attachment percentage, the cultures were incubated for 7h using an orbital shaker at 30°C and 180rpm, and samples were taken at 1, 30, 180, and 420min.

To quantify microbial attachment, 5ml of each sample was centrifuged at 200rpm for 2min (Prism C2500, Labnet International, Edison, NJ, United States), and then, the supernatant was separated from the mineral solid precipitate. Number of attached cells to the minerals were determined by quantitative *real-time* PCR (qPCR) for each species after DNA extraction from mineral pellet. To determine the percentage of attached cells, the number of DNA copies obtained at each time was contrasted with respect to the initial number of DNA copies of each species. Additionally, the total number of planktonic cells present in the supernatant was quantified by direct counting in Neubauer chamber.

### Evaluation of Microbial Attachment on a Pre-colonized Mineral

Attachment tests were carried out with pre-colonization of the mineral. The attachment method consisted of an inoculating process in two consecutive stages, in such a way that the first inoculated strain acted as a pre-colonizer. Both species were tested as pre-colonizers by independent cultures, and thus, when *L. ferrooxidans* DSM 2705^T^ acted as a pre-colonizer, *At. thiooxidans* DSM 14887^T^ was the secondary colonizer and vice versa. This test was carried out using adapted and non-adapted to D-galactose *At. thiooxidans* DSM 14887^T^ and *L. ferrooxidans* DSM 2705^T^.

Experiments were carried out in 250-ml Erlenmeyer flasks with 50ml of Kim medium (without addition of D-galactose, iron, and elemental sulfur) supplemented with 2% w/v of a polymetallic mineral concentrate. To start the first stage of culture, 5·10^7^ cells·ml^−1^ of the pre-colonizing strain were added; after 60min, the supernatant was separated from the mineral by centrifugation at 200rpm and fresh Kim medium was added together with the same number of cells of the second species. The cultures were kept for 3h at 30°C and 180rpm. Samples were taken after 1, 60, 61, 91, and 181min. For each sample, the supernatant was separated by centrifugation at 200rpm for 2min to quantify the total planktonic cells and the solid was recovered for the determination of the number of attached cells by qPCR for each species. To determine the percentage of attached cells, the number of DNA copies obtained at each time was contrasted with respect to the initial number of DNA copies of each species.

### Determination of Microbial Attachment to Mineral by Quantitative Real-Time PCR

DNA extraction from the bacterial cultures was carried out with the DNA easy Ultraclean Microbial Kit (QIAGEN, Hilden, Germany) according to the protocol suggested by the manufacturer, and the quality and concentration of the extracted DNA were verified using a NanoDrop (BioSpec-Nano, Shimadzu Corporation, Kyoto, Japan). Genomic DNA extraction from colonized solid samples was performed with the Power Soil kit (Mobio Laboratories, Carlsbad, CA, United States).

Primers were designed to quantify the presence of *At. thiooxidans* DSM 14887^T^ and *L. ferrooxidans* DSM 2705^T^ (Table 1). For this, the 16S rRNA gene sequence of reference strains was obtained from the NCBI (National Center for Biotechnology Information) databased and used for a sequence alignment using PHYDE software (Phylogenetic Data Editor). Primer sequences of 18–22 nucleotides were selected; the Guanine/Cytosine content and the annealing temperature were verified by means of Oligocalculator software, as well as the quality and specificity by Primer-BLAST simulator. For final verification, a PCR reaction with DNA obtained from each species was carried out under the determined conditions and the resulting PCR product checked on a 1% agarose gel (see Supplementary Figure S2 in Supplementary Material).

**Table 1:** Designed primers for the quantification by qPCR of bacterial species of *At. thiooxidans* DSM 14887^T^ and *L. ferrooxidans* DSM 2705^T^.

Strain	Name	Sequence (5'→3')	Size (pb)
*At. thiooxidans* DSM 14887^T^	*A. thio* 785F *A. thio* 785R	TCT TCG GAT GCT GAC GAG CGS GTT AGB TAC GAC ACT	785
*L. ferrooxidans* DSM 2705^T^	13-L-974F 13-L-1187R	AGTAGGGAACCGAAAGGGGAAAA AGGGCCATGATGACTTGACG (Hedrich, data not published)	250

Using the designed primers (Table 1), a standard threshold cycle curve (C_t_) (10^−2^–10^−8^) was constructed regarding the number of gene copies (Hedrich et al., 2016). All tests were performed in triplicate in a *real-time* PCR equipment (AriaMX, Agilent Technologies, Santa Clara, CA, United States). Reactions were performed in a total volume of 10μl containing 5μl of Brilliant II SYBR Green qPCR Master Mix, 2.7μl of double distilled water, 0.4μl of each primer, 0.5μl of BSA, and 1μl of DNA sample. The conditions of each cycle were initial denaturation at 95°C for 10min, 40 amplification cycles consisting of denaturation at 95°C for 15s and alignment for 1min at 60°C. The melting curve was constructed after reaching the qPCR runs using the following parameters: a cycle of 95°C for 30s and 72°C for 1min followed by increasing the temperature up to 95°C in 0.3°C increments. The efficiency of the amplification was verified for the precision of the tests, and it was determined by calculating the variation in the C_t_ values in the replicated samples and their SD.

### Effect of the Consortia Characteristics on the Solubilization of a Polymetallic Mineral Concentrate

Biooxidation test were developed in 250-ml Erlenmeyer flasks containing Kim medium with 2% w/v of previously sterilized mineral. All the conditions established for the attachment tests of the microbial consortia of the species *L. ferrooxidans* DSM 2705^T^ and *At. thiooxidans* DSM 14887^T^ previously adapted to D-galactose were tested, being inoculated with a total of 1·10^8^ cells·ml^−1^ in the cells proportion previously defined (50:50; 30:70, and 30:70). In the experiments with pre-colonization, the inoculum for each microorganism was 5·10^7^ cells·ml^−1^. The biooxidation tests were made in batch cultures during 15days at 30°C and 180rpm with an initial pH of 1.8. All experiences were contrasted with cells non-adapted to D-galactose that serving as a control and were carried out under the same conditions. The tests were considered concluded when the concentration of Fe^3+^ stopped increasing. In addition, pH and cell number were measured, and the volumetric productivity (Qp) was determined according to Saavedra et al. (2020).

### Analytical Methods

The total number of planktonic cells was quantified by direct counting in a Neubauer chamber (depth of 0.02mm) using a phase-contrast microscope (BA410, Motic, Hong Kong, China). The concentration of ferrous iron and total iron was determined by the ferrozine method (Lovley and Phillips, 1987) and sulfate content by turbidimetry (Bernal, 1980).

## Results

### Adaptation of *At. thiooxidans* DSM 14887^T^ and *L. ferrooxidans* DSM 2705^T^ at Different Concentrations of D-Galactose

**Figure 1:**
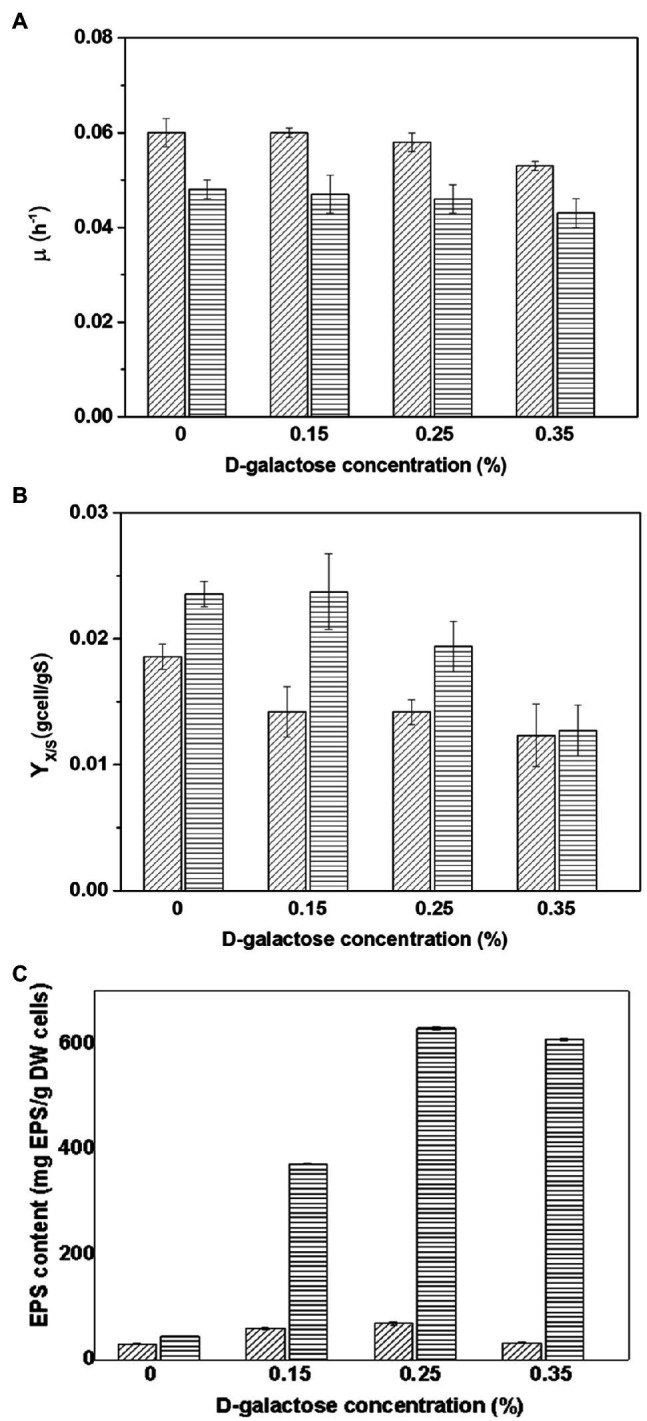
Parameters obtained in the cultures of *Acidithiobacillus thiooxidans* DSM 14887^T^ (diagonal lines) and *Leptospirillum ferrooxidans* DSM 2705^T^ (horizontal lines) adapted to different concentrations of D-galactose. **(A)** Maximum specific growth rate (μ_max_). **(B)** Yield of biomass (Y_X/S_). **(C)** EPS content (mg of EPS by g of DW cells). The data correspond to the mean values of triplicates±SD (*n*=6).

The growth kinetics of *At. thiooxidans* DSM 14887^T^ and *L. ferrooxidans* DSM 2705^T^ were evaluated at different concentrations of D-galactose. Figure 1 resumes the kinetic parameters obtained.

*Acidithiobacillus thiooxidans* DSM 14887^T^ adapted to D-galactose showed a little variation in the maximum specific growth rate at different conditions. The culture without D-galactose (control) and the one with cells adapted to 0.15% of D-galactose are similar and slightly higher compared to the rest of the conditions (Figure 1A). A decrease in pH was also observed during the sulfur oxidation process, thus reaching values of 1.2 (data not shown) resulting from the formation of H_2_SO_4_. The highest biomass yield was obtained for the culture without D-galactose (Figure 1B).

Regarding *L. ferrooxidans* DSM 2705^T^, a higher specific growth rate is also observed in the culture without D-galactose and the culture using cells adapted to 0.15% D-galactose, which are also slightly higher than the values obtained in cultures with cells adapted to 0.25 and 0.35% of D-galactose (Figure 1A). Substrate depletion occurred at approximately 40h for most of the cultures run with adapted cells to D-galactose, except for the case of 0.35%, which continued to grow until approximately 48h.

When the EPS content of *At. thiooxidans* DSM 14887^T^ and *L. ferrooxidans* DSM 2705^T^ adapted to D-galactose was compared, both strains exhibited similar behavior, since the highest concentration of EPS was obtained with both cells adapted to 0.25% of D-galactose (Figure 1C). *Leptospirillum ferrooxidans* DSM 2705^T^ achieved a content of eight times more EPS than *At. thiooxidans* DSM 14887^T^. In both strains, the culture run without D-galactose produced very little EPS, reaching a total of 30.3 and 44.6mg·g^−1^ of dry weight (DW) cell for *At. thiooxidans* DSM 14887^T^ and *L. ferrooxidans* DSM 2705^T^, respectively.

### Microbial Attachment to the Mineral of the Consortia in Proportion 50:50 of *At. thiooxidans* DSM 14887^T^ and *L. ferrooxidans* DSM 2705^T^, Respectively

**Figure 2:**
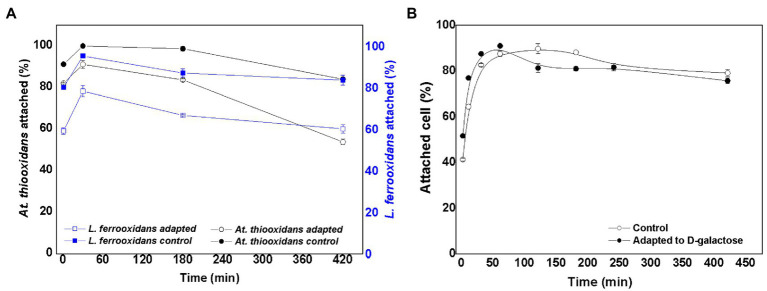
Attachment percentage to the mineral over time for the consortium in proportion 50:50 of *At. thiooxidans* DSM 14887^T^ and *L. ferrooxidans* DSM 2705^T^, respectively. **(A)** Data obtained by qPCR. **(B)** Data obtained by counting total planktonic cells using a Neubauer chamber. The data correspond to the mean values of triplicates±SD (*n*=6).

By mixing equal number of *At. thiooxidans* DSM 14887^T^ and *L. ferrooxidans* DSM 2705^T^, an inoculum was obtained to start the control culture (non-adapted cells) and the culture with adapted cells to D-galactose. In the case of adapted cells, *At. thiooxidans* DSM 14887^T^ and *L. ferrooxidans* DSM 2705^T^ were adapted to 0.15 and 0.25% of D-galactose, respectively. These conditions were selected, because they were the cells that showed the highest attachment percentage as pure cultures (Aguirre et al., 2017, 2018). Figure 2A shows the percentage of attached cells over time for *At. thiooxidans* DSM 14887^T^ and *L. ferrooxidans* DSM 2705^T^ when the consortia were formed in proportion of 50:50 of each species (5·10^7^ cells·ml^−1^ for each strain), both for adapted and non-adapted microorganisms to D-galactose.

In the case of the consortium previously adapted to D-galactose, both species showed an attachment percentage greater than 90% after 60min. On the other hand, in the consortium of non-adapted cells, the attachment of *L. ferrooxidans* DSM 2705^T^ did not exceed 80% in the first hour of the assay; as for the *At. thiooxidans* DSM 148876^T^, it achieved approximately 90% attachment. After 60min, the microorganisms in both consortia slowly dissociated from the mineral, obtaining final attachments (after 7h) ranging from 60 to 84% for both strains in the consortia adapted and non-adapted to D-galactose, respectively. In Figure 2B, a high number of total attached cells to the mineral was observed for the two species that form both consortia (control without adaptation and with previous adaptation to D-galactose). In the case of the control, the attachment percentage reached 87% in the first hour of the experiment, while for the consortium precultured with D-galactose, the attachment percentage was 91% at the same time. After 7h, the cultures with both type of consortia showed an attachment percentage of less than 80%.

### Microbial Attachment to the Mineral of the Consortia Containing a Proportion of 70:30 of *At. thiooxidans* DSM 14887^T^ and *L. ferrooxidans* DSM 2705^T^, Respectively

**Figure 3:**
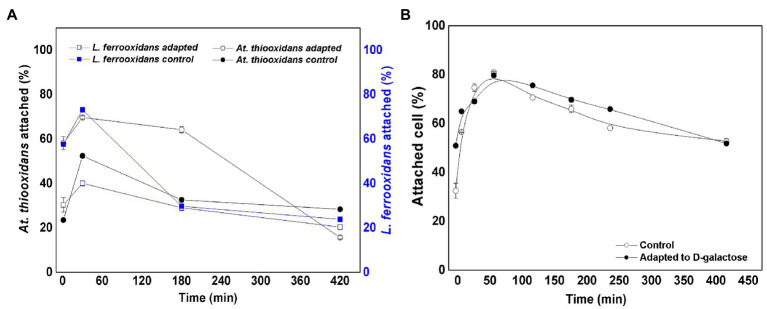
Attachment percentage to the mineral over time for the consortium in proportion 70:30 of *At. thiooxidans* DSM 14887^T^ and *L. ferrooxidans* DSM 2705^T^, respectively. **(A)** Data obtained by qPCR. **(B)** Data obtained by counting of total planktonic cells using a Neubauer chamber. The data correspond to the mean values of triplicates±SD (*n*=6).

Figure 3 shows the variation in the percentage of attached cells over time for *L. ferrooxidans* DSM 2705^T^ and *At. thiooxidans* DSM 14887^T^, when the inoculum concentration was in proportion of 70:30 of *At. thiooxidans* DSM 14887^T^ and *L. ferrooxidans* DSM 2705^T^, respectively, as for the control (non-adapted cells) and the consortium adapted to D-galactose.

For *L. ferrooxidans* DSM 2705^T^, the adaptation to D-galactose has a greater influence on attachment to the mineral, since the attachment percentage exceeds 70% for this strain during the first hour of the experiment (Figure 3A). On the other hand, with *At. thiooxidans* DSM 14887^T^, an opposite behavior is observed. The highest attachment percentage to the mineral occurs in the control culture (non-adapted cells), reaching 69% in the first hour (Figure 3A). After 7h, the attachment percentage of both species is lower, reaching 15 and 30% for the control and adapted *At. thiooxidans* DSM 14887^T^, respectively, while for *L. ferrooxidans* DSM 2705^T^ were obtained 20 and 23% for the control and the adapted, respectively. Figure 3B shows the total attachment percentage of the consortia obtained by counting planktonic cells. It can be observed that during the first hour of the experiment, approximately 80% of the microorganisms were attached to the mineral with respect to the total number of cells inoculated at the start of the experiment (1·10^8^ cells·ml^−1^). Subsequently, the trend of the consortium was like that observed by qPCR, where a decrease in the attachment percentage was observed until it stabilized at approximately 50%.

### Microbial Attachment to the Mineral of the Consortia Containing a Proportion of 30:70 *At. thiooxidans* DSM 14887^T^ and *L. ferrooxidans* DSM 2705^T^, Respectively

**Figure 4:**
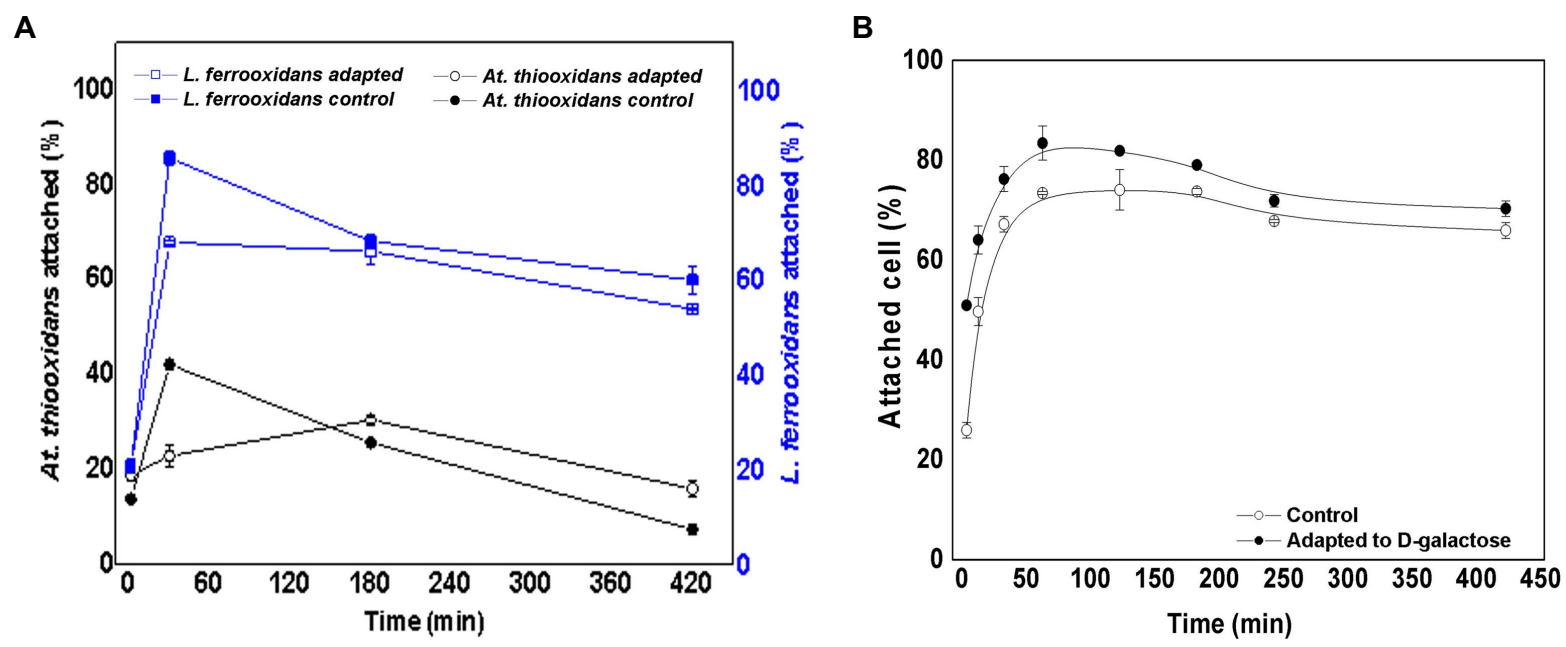
Attachment percentage to the mineral over time for the consortium in proportion of 30:70 of *At. thiooxidans* DSM 14887^T^ and *L. ferrooxidans* DSM 2705^T^, respectively. **(A)** Data obtained by qPCR. **(B)** Data obtained by counting of total planktonic cells using a Neubauer chamber. The data correspond to the mean values of triplicates±SD (*n*=6).

Figure 4 shows the variation in the attachment percentage over time of *L. ferrooxidans* DSM 2705^T^ and *At. thiooxidans* DSM 14887^T^, when the inoculum concentration was in proportion of 30:70 of *At. thiooxidans* DSM 14887^T^ and *L. ferrooxidans* DSM 2705^T^, respectively, as for the control (non-adapted cells) and the consortium adapted to D-galactose.

In the consortium adapted to D-galactose, *L. ferrooxidans* DSM 2705^T^ had a higher attachment percentage (85%) with respect to *At. thiooxidans* DSM 14887^T^, which only reaches 42%. For the control, the trend was similar; however, the attachment percentage was lower for both species, reaching approximately 67% for *L. ferrooxidans* DSM 2705^T^ and 30% for *At. thiooxidans* DSM 14887^T^. Figure 4B shows the total attachment percentage calculated as difference of initial total cells and the remaining planktonic cells. It is observed that approximately 83% of the total cells attach to the mineral when the consortium was adapted to D-galactose, while the control achieved approximately 70% of attachment cells, based on the total cells inoculated at the beginning of the assay (1·10^8^ cells·ml^−1^).

### Microbial Attachment of *L. ferrooxidans* DSM 2705^T^ and *At. thiooxidans* DSM 14887^T^ to Mineral Pre-colonized With *L. ferrooxidans* DSM 2705^T^

**Figure 5:**
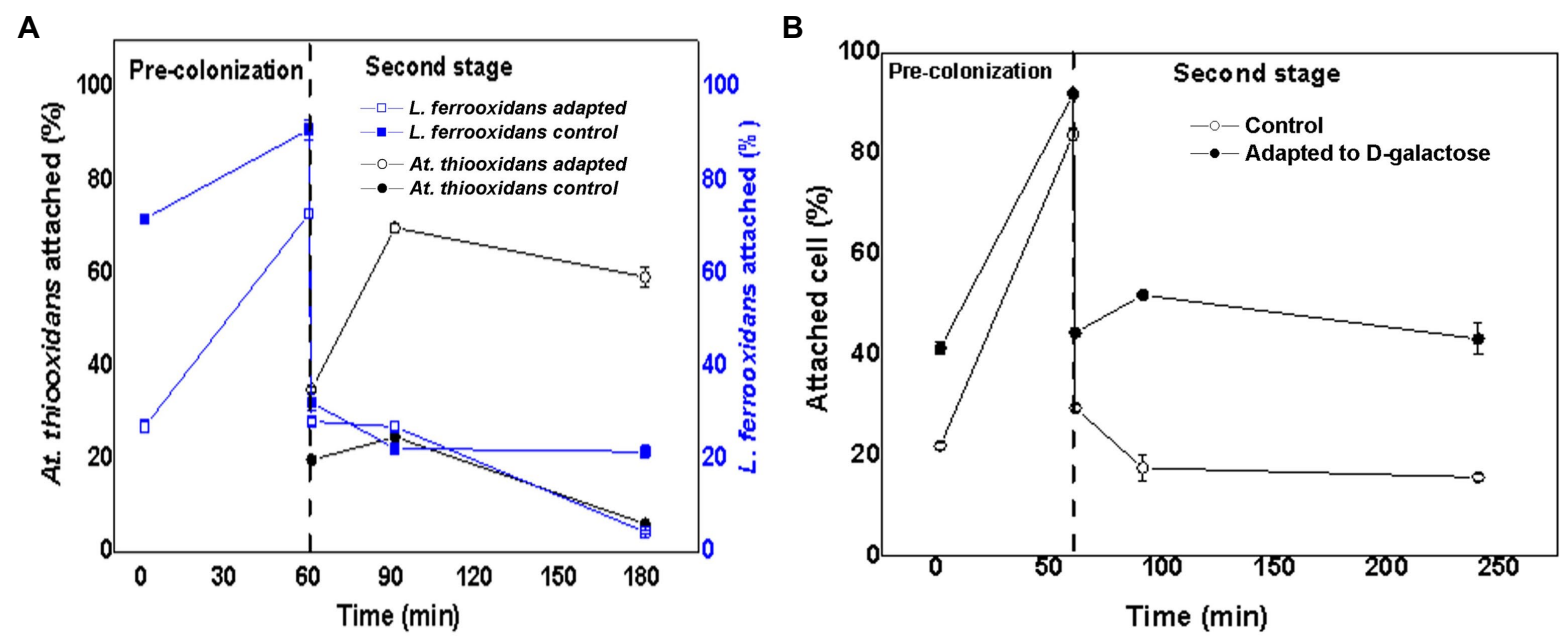
Attachment percentage to the mineral over time of *L. ferrooxidans* DSM 2705^T^ and *At. thiooxidans* DSM 2705^T^ using *L. ferrooxidans* DSM 2705^T^ as a pre-colonizer. **(A)** Data obtained by qPCR. **(B)** Data obtained by counting total planktonic cells using a Neubauer chamber. Measurements after 1, 60, 61, 91, and 181min of inoculation of the first species. The data correspond to the mean values of triplicates±SD (*n*=6).

Figure 5 shows the variation of the attachment percentage over time of *L. ferrooxidans* DSM 2705^T^ and *At. thiooxidans* DSM 14887^T^ to the mineral pre-colonized with *L. ferrooxidans* DSM 2705^T^, for the consortia of non-adapted and adapted cells to D-galactose.

After 60min, *L. ferrooxidans* DSM 2705^T^ reached a higher attachment percentage (88%) when the consortia were adapted, while the control only reached 71% (Figure 5A). After 61min, when the second species (*At. thiooxidans* DSM 14887^T^) was added, the attachment percentage of *L. ferrooxidans* DSM 2705^T^ decreased drastically, both in the control and in the consortium adapted to D-galactose. After 181min, the cells of non-adapted consortium had almost completely dissociated from the mineral, while cells of the adapted consortium remained attached at almost the same percentage as after the inoculation of *At. thiooxidans* DSM 14887^T^.

On the other hand, for *At. thiooxidans* DSM 14887^T^ in the non-adapted consortium, the attachment percentage increased from 35 to 70% after 30min from their inoculation (91min); then, it decreased until reaching 60% at the end of the experience. In the case of the consortia adapted to D-galactose, the attachment percentage was low throughout the experiment; the observed trend was quite similar, but the maximum attachment percentage only reached 24% at 91min.

Figure 5B shows the microbial attachment of total cells adapted and non-adapted to D-galactose acting *L. ferrooxidans* DSM 2705^T^ as a pre-colonizer. After 60min of incubation, a similar attachment percentage was observed compared with the results obtained by qPCR. At minute 61, when the second species (*At. thiooxidans* DSM 14887^T^) was added, it was observed that the global attachment percentage of the culture decreased, being greater for the consortium adapted to D-galactose. After 7h of culture, it was possible to maintain 40% of the attachment.

### Microbial Attachment of *L. ferrooxidans* DSM 2705^T^ and *At. thiooxidans* DSM 14887^T^ to Mineral Pre-colonized With *At. thiooxidans* DSM 14887^T^

**Figure 6:**
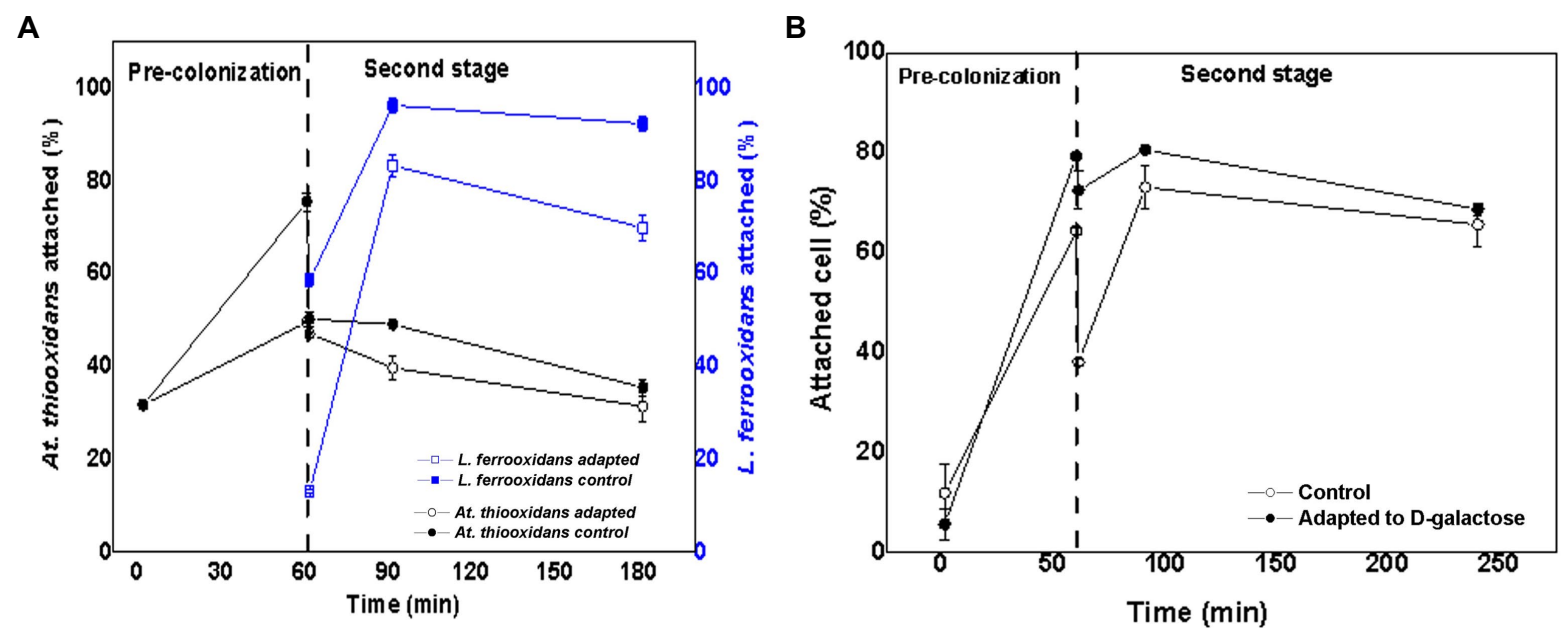
Attachment percentage to the mineral over time of *L. ferrooxidans* DSM 2705^T^ and *At. thiooxidans* DSM 2705^T^ using *At. thiooxidans* DSM 2705^T^ as a pre-colonizer. **(A)** Data obtained by qPCR. **(B)** Data obtained by counting total planktonic cells using a Neubauer chamber. Measurements after 1, 60, 61, 91, and 181min of inoculation of the first species. The data correspond to the mean values of triplicates±SD (*n*=6).

Figure 6 shows the variation of the attachment percentage over time of *L. ferrooxidans* DSM 2705^T^ and *At. thiooxidans* DSM 14887^T^ to mineral pre-colonized with *At. thiooxidans* DSM 14887^T^, for the consortia non-adapted and adapted to D-galactose.

At minute 61, after inoculating *At. thiooxidans* DSM 14887^T^, the attachment percentage of *L. ferrooxidans* DSM 2705^T^ was much higher in the consortium adapted to D-galactose than in the control, which only reached 13% (Figure 6A). The highest attachment percentage was observed at 91min after having inoculated the first species (*At. thiooxidans* DSM 14887^T^), reaching 83 and 96% of attachment for the control culture and the previously adapted, respectively. Finally, at 181min, it was observed that *L. ferrooxidans* DSM 2705^T^ detached itself from the mineral; this phenomenon was more marked for the control.

In the case of *At. thiooxidans* DSM 14887^T^, an attachment percentage of approximately 90% was obtained during the first 60min for the consortium adapted to D-galactose, which contrasts with the control that only achieved 40% of attachment (Figure 6A). At 61min, when *L. ferrooxidans* DSM 2705^T^ had been introduced, *At. thiooxidans* DSM 14887^T^ began to detach. This behavior was similar in the case of the adapted consortium and in the control with cells non-adapted to D-galactose.

Figure 6B shows attachment of total cells for the consortia of non-adapted and adapted cells to D-galactose using *At. thiooxidans* DSM 14887^T^ as a pre-colonizer. Up to minute 60, where only *At. thiooxidans* DSM 14887^T^ was present, the percentage of microbial attachment was like the results obtained from qPCR. At minute 61, when the second species (*L. ferrooxidans* DSM 2705^T^) had been added, the trend of the consortium changed slightly, obtaining a higher attachment percentage with respect to the cultures where the pre-colonizer was *L. ferrooxidans* DSM 2705^T^.

### Comparison of Attachment Percentage of the Consortia on the Surface of the Mineral Particles

**Figure 7:**
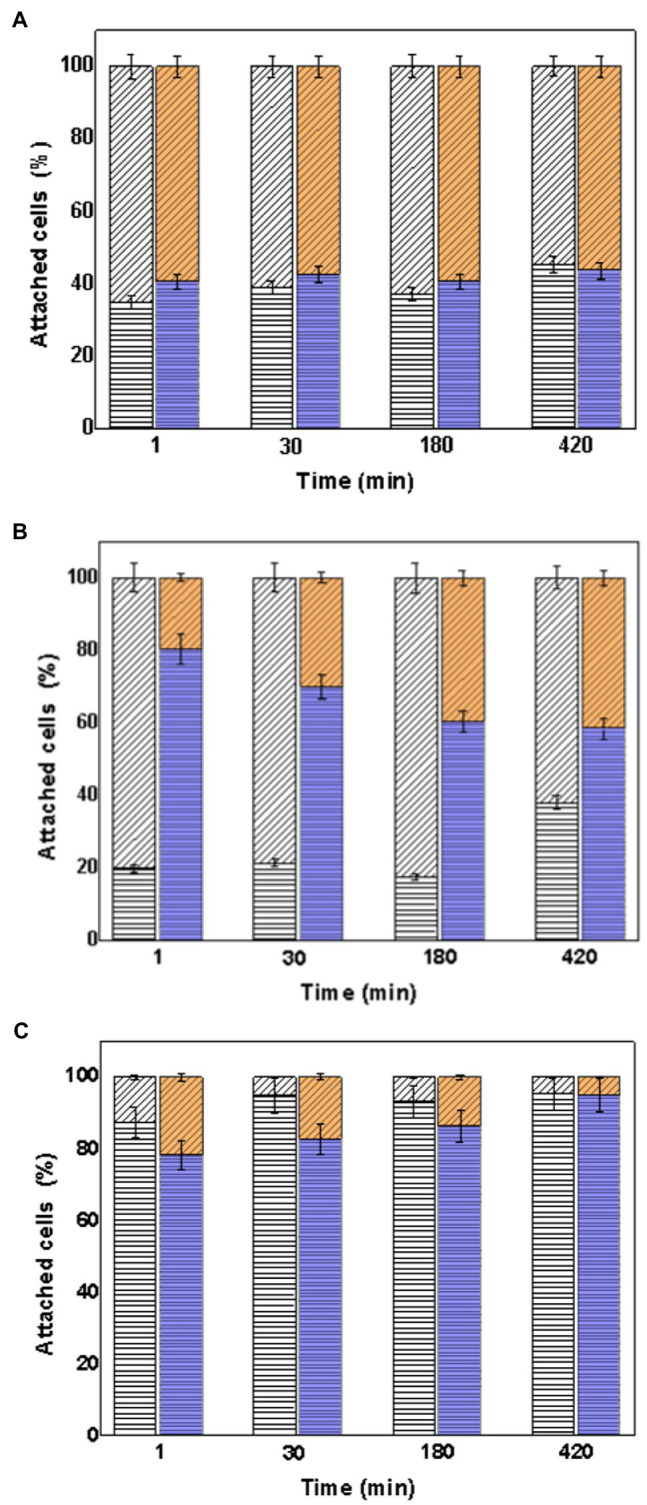
Attachment percentage of the species *At. thiooxidans* DSM 14887^T^ and *L. ferrooxidans* DSM 2705^T^ on the surface of the mineral particles over time. **(A)** Consortia in proportion 50:50 of each specie. **(B)** Consortia in proportion 70:30 of *At. thiooxidans* DSM 14887^T^ and *L. ferrooxidans* DSM 2705^T^, respectively. **(C)** Consortia in proportion 30:70 of *At. thiooxidans* DSM 14887^T^ and *L. ferrooxidans* DSM 2705^T^, respectively. *At. thiooxidans* DSM 14887^T^ (diagonal lines) and *L. ferrooxidans* DSM 2705^T^ (horizontal lines). White Bars represent the control. Color Bars represent consortia adapted to D-galactose. The data correspond to the mean values of triplicates±SD (*n*=6).

It was also determined how to distribute the consortia on the surface of the mineral particles. Figure 7 shows the percentage of attached cells of *At. thiooxidans* DSM 14887^T^ and *L. ferrooxidans* DSM 2705^T^ with respect to the total cells attached to the mineral particles determined by qPCR.

According to Figure 7, the content of *At. thiooxidans* DSM 14887^T^ and *L. ferrooxidans* DSM 2705^T^ was close to 50% in the consortium in proportion 50:50 of both species (Figure 7A). However, the amount of *L. ferrooxidans* DSM 2705^T^ increased in all consortia adapted to D-galactose, even in the consortium formed in a proportion of 70:30 of *At. thiooxidans* DSM 14887^T^ and *L. ferrooxidans* DSM 2705^T^, respectively, (Figure 7B). The highest percentage of the attached cells to mineral of *At. thiooxidans* DSM 14887^T^ was observed in the control consortium 70:30 of *At. thiooxidans* DSM 14887^T^ and *L. ferrooxidans* DSM 2705^T^.

**Figure 8:**
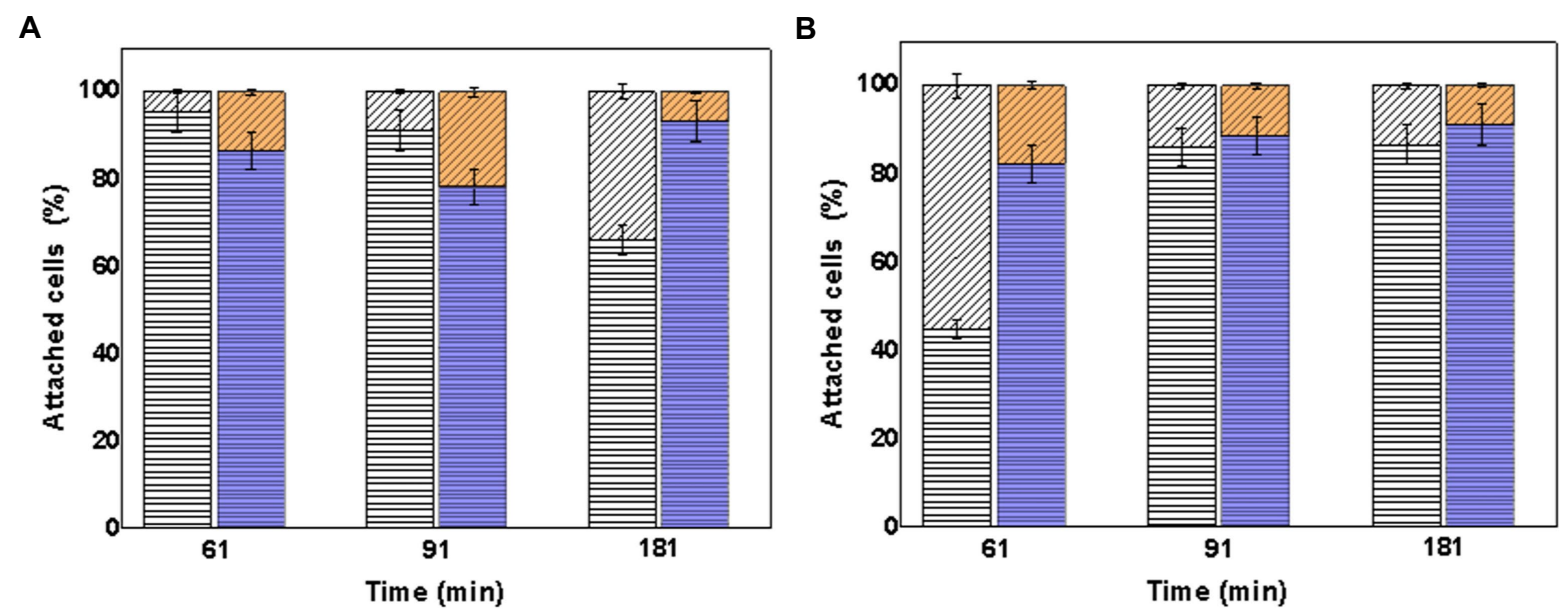
Attachment percentage of the species *At. thiooxidans* DSM 14887^T^ and *L. ferrooxidans* DSM 2705^T^ on the surface of the mineral particles determined in the time. **(A)** Using *L. ferrooxidans* DSM 2705^T^ as a pre-colonizer. **(B)** Using *At. thiooxidans* DSM 2705^T^ as a pre-colonizer. Measurements at 61, 91, and 181min from the inoculation of the first species. *At. thiooxidans* DSM 14887^T^ (diagonal lines) and *L. ferrooxidans* DSM 2705^T^ (horizontal lines). White Bars represent the control. Color Bars represent consortia adapted to D-galactose. The data correspond to the mean values of triplicates±SD (*n*=6).

In the same way, it was also determined from the total number of attached cells of the consortia to the mineral how much corresponds to each species. Figure 8 shows the percentage of attached cells of each species (*At. thiooxidans* DSM 14887^T^ and *L. ferrooxidans* DSM 2705^T^), with respect to the total cells attached to the mineral particles determined by qPCR using one species as pre-colonizer.

The content of *L. ferrooxidans* DSM 2705^T^ was in higher percentage in all consortia, mainly in which this microorganism acted like a pre-colonizer. The highest percentage of attached cells to mineral of *At. thiooxidans* DSM 14887^T^ was observed in the non-adapted to D-galactose consortium where this microorganism was used as pre-colonizer.

### Effect of the Consortia Characteristics on the Biooxidation of a Polymetallic Mineral Concentrate

Table 2 shows pH variation and the overall volumetric productivity (Qp) in the biooxidation of a polymetallic mineral concentrate carried out by consortia of different characteristics. According to these results, the consortia of non-adapted cells to D-galactose presented the lowest pH variation, being the consortia in proportion of 30:70 of *At. thiooxidans* DSM 14887^T^ and *L. ferrooxidans* DSM 2705^T^, respectively, the combination that obtained the lowest differential with a ΔpH value of 0.12. On the other hand, the greater pH differential was observed in the consortia adapted to D-galactose, especially in the proportion of 50:50 of both species. ΔpH value was 0.38, reaching pH of 1.4 at the time in which biooxidation tests were completed. At the same time, this consortium registered a maximum level of 9.7mgL^−1^ h^−1^ of overall volumetric productivity of Fe^3+^. On the other hand, the one with the lowest overall volumetric productivity of Fe^3+^ was the consortium formed by *L. ferrooxidans* DSM 2705*T* and *At. thiooxidans* DSM 14887^T^ non-adapted to D-galactose, using *At. thiooxidans* as a pre-colonizer, after a period of 15days of biooxidation.

**Table 2:** pH variation and overall volumetric productivities of the different consortia formed by the species *At. thiooxidans* DSM 14887^T^ and *L. ferrooxidans* DSM 2705^T^.

Consortium	ΔpH	Qp [mg·L^−1^·h^−1^]	
		Fe ^3+^	SO_4_^2−^
50:50 of *At. thiooxidans* DSM 14887^T^ and *L. ferrooxidans* DSM 2705^T^ (control)	0.24	2.8	6.6
50:50 of *At. thiooxidans* DSM 14887^T^ and *L. ferrooxidans* DSM 2705^T^ (adapted)	0.38	9.7	11.7
70:30 of *At. thiooxidans* DSM 14887^T^ and *L. ferrooxidans* DSM 2705^T^ (control)	0.23	3.7	6.0
70:30 of *At. thiooxidans* DSM 14887^T^ and *L. ferrooxidans* DSM 2705^T^ (adapted)	0.36	9.1	5.7
30:70 of *At. thiooxidans* DSM 14887^T^ and *L. ferrooxidans* DSM 2705^T^ (control)	0.12	4.7	4.4
30:70 of *At. thiooxidans* DSM 14887^T^ and *L. ferrooxidans* DSM 2705^T^ (adapted)	0.32	3.6	6.5
Using *L. ferrooxidans* DSM 2705^T^ as a pre-colonizer (control)	0.18	3.1	4.9
Using *L. ferrooxidans* DSM 2705^T^ as a pre-colonizer (adapted)	0.30	5.4	6.1
Using *At. thiooxidans* DSM 2705^T^ as a pre-colonizer (control)	0.23	2.0	8.9
Using *At. thiooxidans* DSM 2705^T^ as a pre-colonizer (adapted)	0.37	7.9	9.6

Standard error (*n*=6) ≤5%.

The overall volumetric productivity of SO_4_^2−^ was low in consortia where the proportion of *A. thiooxidans* DSM 14887^T^ was lower, such was the case of the consortium formed in proportion of 30:70 of *At. thiooxidans* DSM 14887^T^ and *L. ferrooxidans* DSM 2705^T^ non-adapted to D-galactose. In the same way, the highest overall volumetric productivity of SO_4_^2−^ occurred in the consortium formed in proportion 50:50 of *At. thiooxidans* DSM 14887^T^ and *L. ferrooxidans* DSM 2705^T^ adapted to D-galactose with a value of 11.7mgL^−1^ h^−1^ after 15days of biooxidation.

## Discussion

The tests carried out with mixed cultures of *L. ferrooxidans* DSM 2705^T^ and *At. thiooxidans* DSM 14887^T^ indicate that the combination of strains influences the binding capacity of each one on sulfide minerals. The results indicate that in most of the combinations of *L. ferrooxidans* DSM 2705^T^ and *At. thiooxidans* DSM 14887^T^, bacteria mutually influence the mineral binding capacity, while the use of inducers of EPS synthesis, in this case D-galactose, has a potentiating effect on this binding. Similar results were obtained by other authors using different inducers like UVA radiation which promoted the formation of EPS and cell attachment (Amar et al., 2021).

In the case of *L. ferrooxidans* DSM 2705^T^ when combined with *At. thiooxidans* DSM 14887^T^, the binding to sulfides is lower compared to pure cultures, which reach an attachment percentage greater than 90% (Aguirre et al., 2018). However, the attachment of *At. thiooxidans* DSM 14887^T^ tends to increase in other combinations. Thus, for example, *At. thiooxidans* DSM 14887^T^ was the strain with the highest attachment percentage in the consortium with proportion 50:50 and showed the highest attachment in the consortium with proportion 70:30 of *At. thiooxidans* DSM 14887^T^ and *L. ferrooxidans* DSM 2705^T^, respectively. When used as a pre-colonizer, *L. ferrooxidans* DSM 2705^T^ showed reduced overall attachment capacity in mixed cultures compared to pure cultures. The results suggest that *At. thiooxidans* DSM 14887^T^ attach faster and in greater proportion to the mineral when it is previously adapted to D-galactose. For all cases, it is important to highlight that the moment with the highest attachment percentage occurred in the first hour of the test and then declined over time.

With respect to the variation of the attachment percentage over time with different proportions of each species, when the microbial consortium was tested in a proportion of 70:30 of *At. thiooxidans* DSM 14887^T^ and *L. ferrooxidans* DSM 2705^T^ (Figure 3A) showed a high attachment percentage of the first species, without neglecting that the second one had an attachment percentage of 70±5.7% when the consortium was previously adapted to D-galactose. As for the control, both species follow the same trend; however, the attachment percentage in general is lower. Respect to the attachment percentage of *At. thiooxidans* DSM 14887^T^, it seems that when this species is initially present in a greater number of cells and forming a thin layer of biofilm, this would allow that the other species to easily attach to the mineral, without having a greater impediment to the attachment of *L. ferrooxidans* DSM 2705^T^. On the other hand, when the proportion of *At. thiooxidans* DSM 14887^T^ is lower (Figure 4A), it seems that *L. ferrooxidans* DSM 2705^T^ occupies almost all the active sites of the mineral; in addition, having a greater amount of EPS (Figure 1C), it prevents *At. thiooxidans* DSM 14887^T^ of occupying available active sites for its attachment (Florian et al., 2010; Florian, 2012). Likewise, it is known the existence of a secretion of microbial lipids called surfactant microbial compounds (SAC) that are involved in the alteration of hydrophobic surfaces. SACs secreted by bacteria into the surrounding media may be responsible for a microbially created conditioning film at an interface (Neu, 1996; Zhang et al., 2015; Castro et al., 2016). One of the first examples was reported for a species of *Acidithiobacillus* spp.; some compounds of this surface may be involved in the initial stages of attachment to hydrophobic surfaces (Jones and Starkey, 1961; Georgiou et al., 1992; Chandrawati and Shagufta, 2007). The presence of microbial surfactant compounds can also inhibit bacterial attachment to solid surfaces. Another cause for the decreased attachment of at least one of the strains may be competition for attractive (usable) surface areas by bacteria.

Regarding the attachment percentage of total cells when the proportion of each species is varied (Figures 2B, 3B, 4B), it is important to remark that the consortium adapted to D-galactose with a 50:50 proportion presents a higher attachment percentage compared to consortium non-adapted to D-galactose. The tests also show a similar trend to those carried out using qPCR, accounting for the behavior of the microbial species. It is interesting to highlight that the maximum attachment percentage developed up to 2h test; after such point, it slowly began to decrease.

There are studies in which the importance of EPS has been demonstrated for microbial attachment to sulfide minerals; however, little is known about the interactions generated among various species and their EPS on attachment to minerals sulfides and in turn the effect it can have on the biooxidation of sulfides. From the tests using pre-colonization with non-adapted and adapted cells, a behavior like those described in the previous cases was observed with respect to the attachment percentage of a consortium. Thus, for example, in Figure 5, it is observed that *L. ferrooxidans* DSM 2705^T^ when used as a pre-colonizer after 1h of incubation, its attachment percentage is over 70±1% for the control and 90±3.25% for the consortium adapted to D-galactose, results that agree with the previously discussed pure culture data (Florian et al., 2010; Florian, 2012; Aguirre et al., 2018).

When pre-colonization started with *At. thiooxidans* DSM 14887^T^, a similar behavior of this species was observed after 1h of incubation of non-adapted and adapted to D-galactose consortium. After minute 61, when the second species was inoculated, their attachment percentage decreased. In this case, the attachment percentage of *L. ferrooxidans* DSM 2705^T^ was higher in non-adapted and adapted to D-galactose consortium, the last one being higher than the control. Apparently, the forces involved in the EPS of *At. thiooxidans* DSM 14887^T^ are weak. In addition, the amount of EPS formed was much lower compared with *L. ferrooxidans* DSM 2705^T^ (Gilbert et al., 1993).

In the same way, the amount of each microorganism attached to the mineral particles was investigated in function of the total amount of microorganisms that only were attached to the mineral (Figure 7). When the strains are in a 50:50 ratio of each species, *At. thiooxidans* DSM 14887^T^ achieves a higher attachment with respect to *L. ferrooxidans* DSM 2705^T^ in the control; however, when they are previously adapted to D-galactose, the attachment of *At. thiooxidans* DSM 14887^T^ decreases, while that of *L. ferrooxidans* DSM 2705^T^ increases.

This phenomenon can be explained since in the consortium formed in a 50:50 ratio of each species, the amount of *L. ferrooxidans* DSM 2705^T^ cells with EPS was lower compared to the consortium previously adapted to D-galactose, whose amount of microorganism with EPS was much greater, occupying most of the active sites of the mineral. Additionally, a strong EPS formation has already been described in case of atomic force microscopy (AFM) and epifluorescent microscopic (EFM) studies of *Leptospirillum* spp. (Noël, 2010; Florian et al., 2011; Florian, 2012). These results are agreed with Liu et al. (2016) who tested a binary mixture of *Leptospirillum ferriphilum* and *Acidithiobacillus* showed the same proportion that each represented 50% at final equilibrium stage. *L. ferriphilum* and *At. caldus* can attach on chalcopyrite simultaneously and performed mutual non-interference in their adsorption to chalcopyrite. The high proportion of *L. ferriphilum* at firstly stage was due to the higher affinity and EPS, which benefited the preferential attachment of *L. ferriphilum*.

Likewise, when the microorganisms are in the consortium in a 70:30 ratio of *At. thiooxidans* DSM 14887^T^ and *L. ferrooxidans* DSM 2705^T^, respectively, in the control is observed that the predominant species is *At. thiooxidans* DSM 14887^T^. In the consortium in the same proportion (70:30) but adapted to D-galactose, it can be observed that the species with the highest attachment is *L. ferrooxidans* DSM 2705^T^. Finally, when the proportion of the consortium is reversed, both the control and the one previously adapted to D-galactose a high attachment of *L. ferrooxidans* DSM 2705^T^ is observed.

In the case of consortia using pre-colonizer (Figure 8), similar phenomena happened. There was more attachment percentage of *L. ferrooxidans* DSM 2705^T^ when it was used as pre-colonizer. In the other hand, when *At. thiooxidans* DSM 14887^T^ was used as pre-colonizer, and once *L. ferrooxidans* was inoculated, it started to predominate and displaced *At. thiooxidans* DSM 14887^T^, also reaching a high attachment percentage to the mineral surface. Again, this behavior can be explained because of the higher amount of its cells EPS and probably stickier than the EPS of *At. thiooxidans* DSM 14887^T^.

On the other hand, bacterial oxidation of mineral sulfides is a process that is influenced by several factors, such as temperature, pH, availability of O_2_ and CO_2_, the presence of toxic compounds, microbial species, among others. Hence, all the consortia previously established to carry out the attachment tests were investigated as biooxidant agents. It is known that bacterial attachment is important for biooxidation processes, affecting the efficiency of dissolution of sulfur minerals. According to Makaula et al. (2020) is recognized that the effective colonization of the mineral ore bed for heap bioleaching impacts both the start-up time of the heap and its performance.

Table 2 shows the pH differential due to its decrease during the biooxidation process of the concentrate of polymetallic sulfide mineral. It should be noted that these results represent an indirect measure of the oxidation of the minerals, since it is indicative that H_2_SO_4_ was generated as a product. The highest pH differential occurs when both species are in a proportion of 50:50 and when *At. thiooxidans* DSM 14887^T^ is used as a pre-colonizer, either for non-adapted or for adapted to D-galactose consortium. On the other hand, the high overall volumetric productivity of Fe^3+^ was determined with consortium previously adapted to D-galactose in proportion 50:50 of each species, that is, those with the highest content of both species. It should also be noted that the high concentrations of Fe^3+^ at the beginning of the biooxidation could have influenced the process, since this consortium probably accumulated a greater amount of iron within their EPS, which in turn allowed them to have a greater dissolution of the minerals even with respect to controls without prior adaptation to D-galactose (Sand et al., 1995).

A careful analysis that combines the results of the adhesion tests and the biooxidation experiments of the sulfide minerals would provide more in-depth information that evidences a connection between the initial bacterial binding and the mineral solubilization, since the cultures that contained greater microbial attachment of both species adapted to D-galactose were those that provided a greater dissolution of sulfur minerals. The presence of EPS on the surface of bacteria and mineral plays an important role on bioleaching (Fang et al., 2021; Ye et al., 2021).

## Conclusion

The proportion of the species and the inoculation order of the strains in the consortium together with the EPS induction with D-galactose affected the cellular attachment percentage to the mineral. The highest attachment percentage of the microbial community was obtained when *L. ferrooxidans* DSM2705^T^ and *At. thiooxidans* DSM 14887^T^ were inoculated in a proportion 50:50 simultaneously and previously adapted to D-galactose. Given the high amounts of EPS in planktonic cells of the acidophilic microorganisms studied, there was an increase in the microbial attachment percentage, which would make possible to accelerate the biooxidation of sulfides minerals.

The proportion of the species and the order of inoculation of the microbial consortium influenced the biooxidation of a polymetallic mineral concentrate, obtaining the highest volumetric productivity of iron and sulfate when *L. ferrooxidans* DSM2705^T^ and *At. thiooxidans* DSM 14887^T^ were inoculated in a proportion of 50:50 simultaneously and previously adapted to D-galactose. Is important consider that better volumetric productivities values (ferric and sulfate) were obtained when both species were inoculated simultaneously, mainly for Fe^3+^, and when the attachment cell percentage to the mineral particles was closer. In the case of sulfate, high volumetric productivity values occurred with consortia adapted and non-adapted to D-galactose, meaning a certain independence on the amount of accumulated EPS.

## Data Availability Statement

The original contributions presented in the study are included in the article/Supplementary Material, further inquiries can be directed to the corresponding author.

## Author Contributions

JG proposed and designed the idea and the study, provided the facilities, funding, revised, commented, and contributed to writing the manuscript. PA proposed and designed the idea and the study, performed the experiments, collected, processed, and analyzed data, was involved in the study design, and contributed to writing the manuscript. AS, EM, and KG were involved in data analysis, discussed the results, and contributed to writing the manuscript. SH was involved in data analysis, discussed the results, and contributed to revision of the manuscript. All authors contributed to the article and approved the submitted version.

## Funding

This work was supported by the School of Biochemical Engineering (Pontificia Universidad Católica de Valparaíso-Chile) and Universidad Técnica Particular de Loja (Loja-Ecuador).

## Conflict of Interest

The authors declare that the research was conducted in the absence of any commercial or financial relationships that could be construed as a potential conflict of interest.

## Publisher’s Note

All claims expressed in this article are solely those of the authors and do not necessarily represent those of their affiliated organizations, or those of the publisher, the editors and the reviewers. Any product that may be evaluated in this article, or claim that may be made by its manufacturer, is not guaranteed or endorsed by the publisher.
