# Supplementary material for: Sticky Bacteria: Understanding the Behavior of a D-Galactose Adapted Consortium of Acidophilic Chemolithotroph Bacteria and Their Attachment on a Concentrate of Polymetallic Mineral
Source: Front Microbiol. 2021 Oct 21;12:767639. doi: 10.3389/fmicb.2021.767639 (PMC8566890; doi:10.3389/fmicb.2021.767639)
Supplement: Supplementary file 1 [file Table_1.DOCX]

Supplementary Material

##
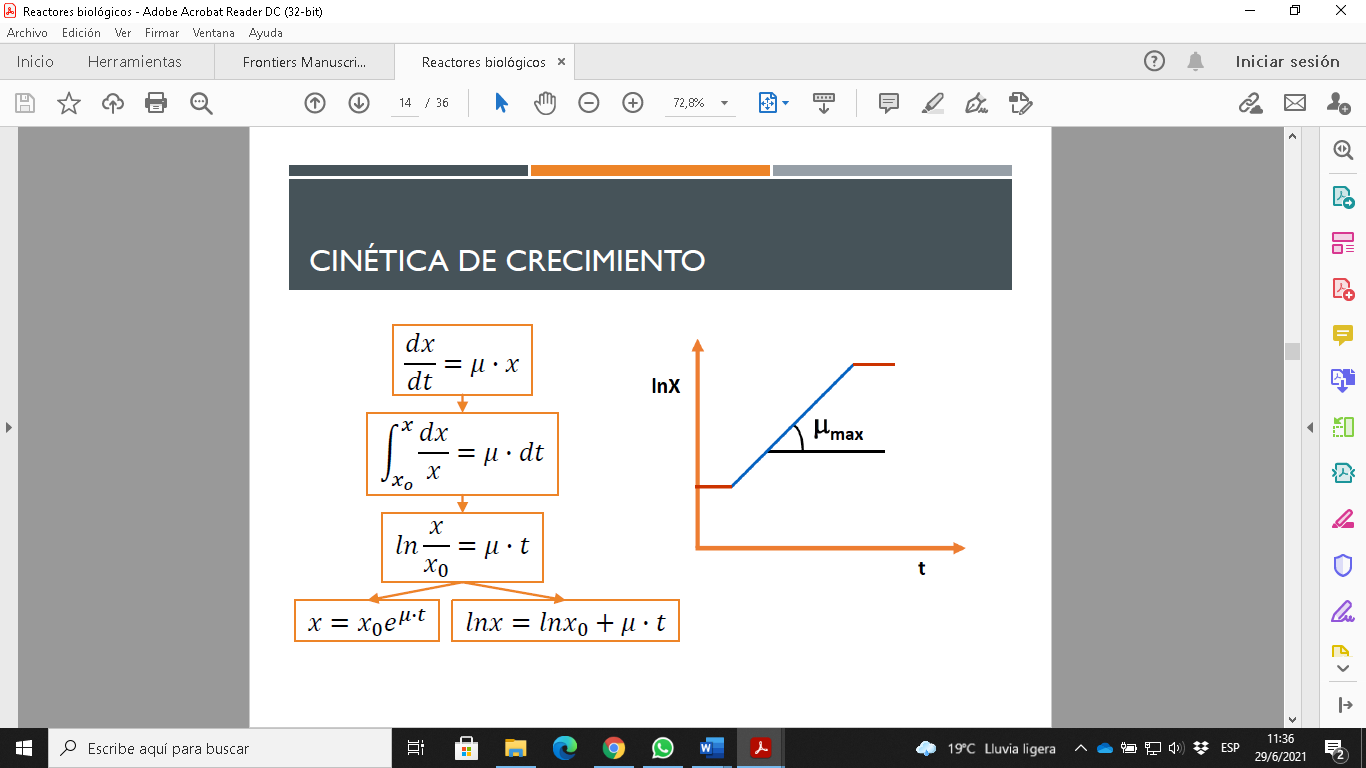
 ****Determination of the maximum specific growth rate (µ_max_)****

$$\frac{dx}{dt}= \mu\cdot x$$

$$\int_{X_{0}}^{X} \frac{dx}{x}=\mu\cdot dt$$

$$ln\frac{x}{x_{0}}= \mu\cdot t$$

$$\ln x= \ln x_{0}+\mu\cdot t$$

- **The cell count data (N) are plotted in time on a semi-logarithmic scale. The slope obtained by linear regression in the exponential growth phase corresponds to the maximum specific growth rate.**
- **The following figure shows the maximum specific growth rate of the species *L. ferrooxidans* DSM 2705^T^ previously adapted to 0.35% galactose in the Kim culture medium modified at pH 1.3: 30 ° C and 180 rpm. The same process was carried out for the species *At. thiooxidans* DSM 14887^T^.**


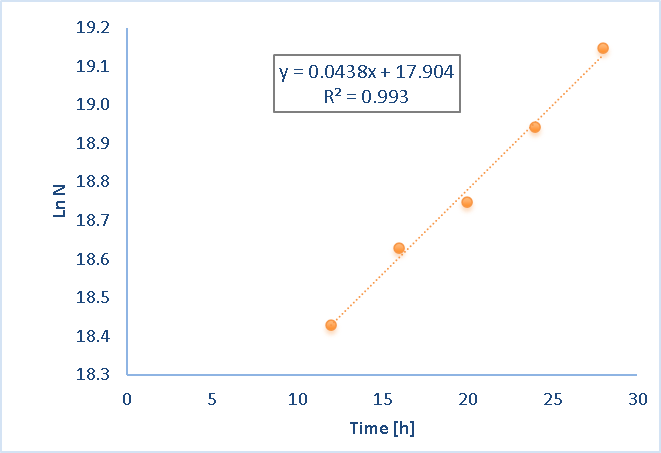


Figure S.1. ****Determination of µ_max_ of the microbial strain *L. ferrooxidans* DSM 2705^T^ previously adapted to 0.35% galactose****

# ****Determination of the biomass yield obtained from the consumption of ferrous ion (****$\boldsymbol{Y}_{\frac{\boldsymbol{x}}{\boldsymbol{s}}}$****)****


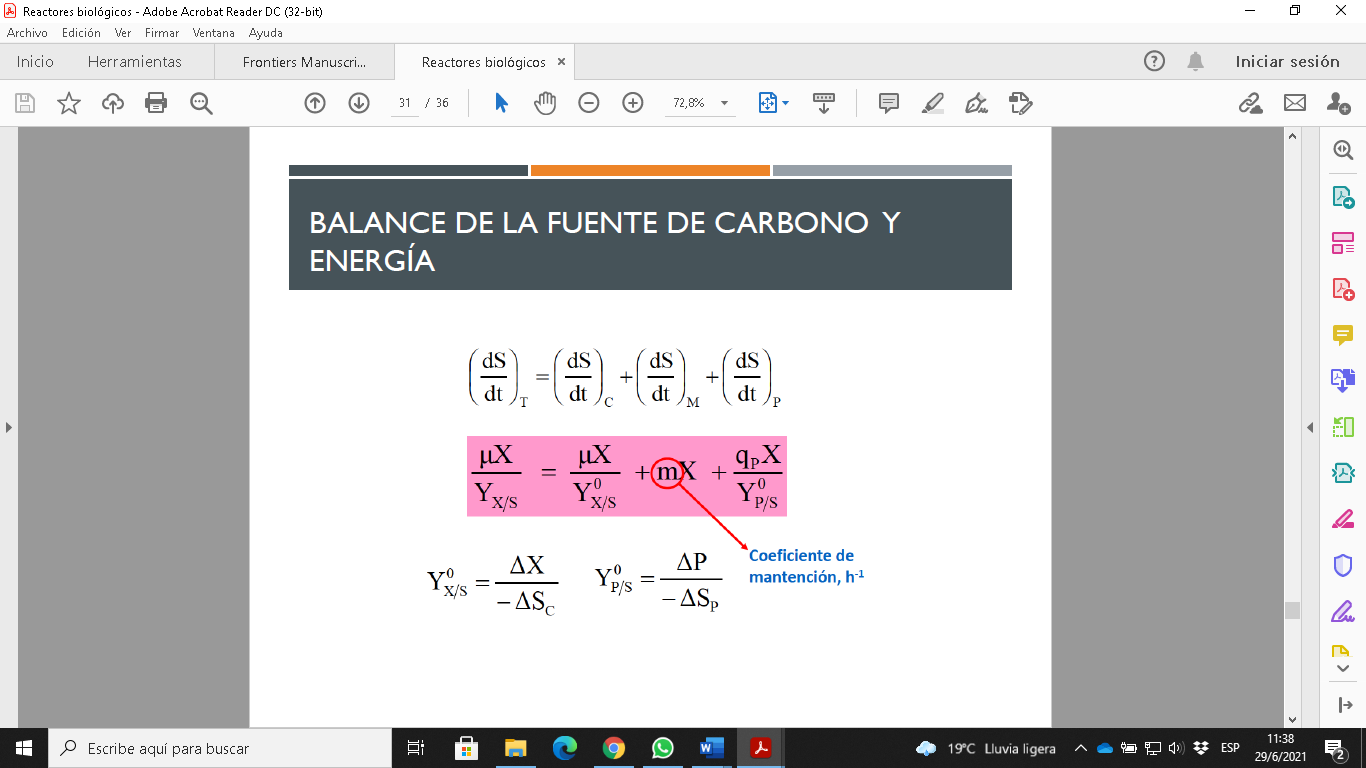

$$Y_{\frac{x}{s}}= \frac{X_{protein final}-X_{protein initial}}{-({Fe}_{final}^{2+}-{Fe}_{initial}^{2+})} x \frac{100}{44}$$

**Units: gcel/g Fe ^2+^**

## ****Specificity of the designed primers for species *Acidithiobacillus thiooxidans* DSM 14887^T^ and *Leptospirillum ferrooxidans* DSM 2705^T^****

**
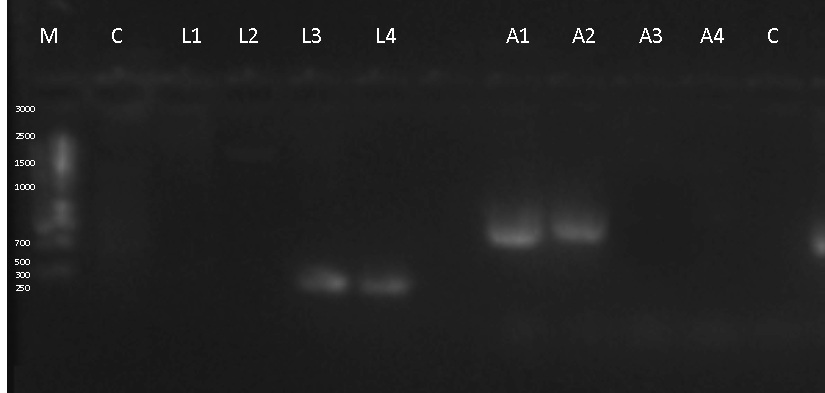
**

- **Figure S2**.  **Agorose gel electrophoresis for verify the specificity of the designed primers for specie *Acidithiobacillus thiooxidans* DSM 14887^T^ y *Leptospirillum ferrooxidans* DSM 2705^T^ M: Ladder 1Kb; C: Negative Control; L: Specific primers for *Leptospirillum ferrooxidans* DSM 2705^T^*^;^* A: Specific primers for *Acidithiobacillus thiooxidans* DSM 14887^T^**
- **1. Sample of *A. thiooxidans* DSM 14887^T^**
- **2. Positive control of *A. thiooxidans* DSM 14887^T^**
- **3. Sample of *L. ferrooxidans* DSM 2705^T^**
- **4. Positive control *L. ferrooxidans* DSM 2705^T^**
